# Building a healthy lifestyle: the role of emotional intelligence among Italian university students

**DOI:** 10.1038/s41598-023-44141-3

**Published:** 2023-10-17

**Authors:** Francesca Licata, Riccardo Maruca, Emma Antonia Citrino, Aida Bianco

**Affiliations:** 1 Department of Health Sciences, School of Medicine, University of Catanzaro “Magna Græcia”, Viale Europa, 88100 Catanzaro, Italy; 2 Department of Medical and Surgical Sciences, School of Medicine, University of Catanzaro “Magna Græcia”, Viale Europa, 88100 Catanzaro, Italy

**Keywords:** Psychology and behaviour, Health care, Disease prevention, Public health

## Abstract

Given the numerous evidence demonstrating the influence of emotions in engaging risky behaviors, it seems inevitable to consider new approaches that promote healthy lifestyles. This study examines the relationship between emotional intelligence (EI) and unhealthy lifestyles among undergraduate university students in Southern Italy, since a correlation between EI and harmful health behaviors has been postulated. The present cross-sectional study was conducted among over 18-year-old university students using an online, anonymous, self-administered questionnaire. Socio-demographic characteristics, tobacco use, nicotine dependence, alcohol consumption, and skipping breakfast were investigated. Nearly a third of the sample were current smokers (30.9%). Problematic drinking was shown in 9.9% of the students. Almost one-fourth (23.1%) reported breakfast skipping ≥ 3 days a week. Emotional clarity and total EI scores were significantly lower in current smokers with moderate/high nicotine dependence. Problematic drinking revealed lower emotional clarity and total EI scores. Breakfast skippers showed lower emotional attention and total EI scores. The interconnectedness of unhealthy behaviors and the potential for one behavior to lead to or predict another were  also shown. The study findings provide useful insights to develop evidence-based strategies to empower the young adults to choose a health-promoting lifestyle. The figures suggest that emotional learning interventions could support this goal.

## Introduction

Noncommunicable diseases (NCDs) cause 74% of global fatalities, with tobacco and alcohol use being major risk factors^1^. The World Health Organization (WHO) has identified a set of actions called “best buys” to achieve global targets for tobacco and alcohol use reduction, including increasing excise taxes and prices on tobacco products and alcoholic beverages and enacting comprehensive bans on tobacco and alcohol advertising, promotion, and sponsorship^2^. Italy ranks in the top 20 countries for implementing WHO-recommended NCDs prevention policies^3^, but occasional and off-meal alcohol consumption has increased in all age groups of the Italian population over the past decade^4^, and the number of smokers has returned to increase for the first time since 2009^5^.

Scientific literature shows that risky habits usually start during adolescence^6–8^. Nevertheless, they could exacerbate and become permanent between the ages of 18 and 30, when young adults experience a significant amount of stress^9^. This transition period from adolescence to young adulthood is critical, as it often involves major life changes such as starting university, living independently, or forming new relationships^10–12^. These difficulties may result in anxiety, loneliness, pressure to perform well academically, and self-doubt; these emotions might become more intense also due to the competitive nature of university settings. As a result, all of these stressors have the potential to have long-term detrimental effects on people’s health and well-being. The burden of trying to balance social obligations and academic responsibilities can induce unhealthy coping strategies, feeding a cycle of stress and harming general well-being. The transition from high school to university also represents a critical period for weight and fat gain^13^. Several studies^14,15^ have highlighted that university students, especially those living away from the family home, have moved away from healthy eating patterns, particularly in Mediterranean countries^16^. Low consumption of fruits and vegetables, excessive consumption of fast food, low consumption of dairy products, and skipping meals are examples of unhealthy eating habits among university students^17^. Breakfast is the most commonly skipped meal for this age group^18^, and this unhealthy dietary practice at a later time is associated with several risk factors for cardiovascular disease^19^ and stress^20^.

It has been demonstrated that the leading causes of morbidity and death today are related to unhealthy lifestyles and chronic stress^21,22^. The fact that to make and to motivate a decision (e.g. engagement in risky behaviors) are deeply affected by emotions^23,24^, makes it imperative to consider this topic in the design of interventions to promote healthy lifestyles. Emotional intelligence (EI), defined as a set of skills that enable people to accurately identify and interpret their own emotions and those of others and use this information to regulate their behavior in social contexts, plays a role as a moderator of the relationship between stress and psychological health. Higher EI scores were discovered to be associated with considerably less reactivity to stress on both a psychological and biological level^25^. A recent meta-analysis supports the existence of a correlation between EI and harmful health behaviors, such as substance abuse, risky sexual behavior, and excessive alcohol consumption^26^. Given that, do higher EI abilities help people to adopt and maintain a healthy lifestyle? We hypothesized that there is a relationship between low EI levels and  the tendency to engage in risky behaviors during university, since EI might enhance emotional control which would lower the chance of engaging in risky behaviors. Therefore, the primary purpose of the present study was to examine the relationship between EI and selected unhealthy lifestyles among undergraduate university students in Southern Italy. The secondary aim was focused on obtaining an overview of distribution of smoking habits and nicotine dependance, alcohol consumption, and breakfast skipping within the sample.

## Materials and methods

### Study design

A cross-sectional study was conducted between the 13th and 28th of February 2023 at the “Magna Græcia” University of Catanzaro, which is located in the Southern part of Italy. The study complies with the STROBE requirements^27^.

### Participant recruitment and sampling

The criteria of eligibility for the study were: (i) being over 18 years old, and (ii) being registered as an undergraduate student at the university. Those who declined to give informed consent were excluded from the study. All the eligible students received an institutional email with a link to an anonymous online survey. Students could agree to participate in the study by checking the box at the bottom of the personal data treatment information sheet on the first page of the online questionnaire. There was also an introductory line that served as an explanation of the study’s goals, clarifying that there was no obligation to complete the questionnaire, and assuring respondents that the data collected would be kept confidential. The questionnaire was safely kept and moved to a password-protected and encrypted computer; there were no identifiers linking students to it. The questionnaire could only be filled out once for each electronic device in an effort to prevent repetitive responses.

### Questionnaire design

The survey was divided into 5 sections. The first section (1) of the questionnaire contained 4 questions to explore social and demographic characteristics. The second section (2) measured EI levels through the Trait Meta-Mood Scale (TMMS). In the third section (3), tobacco use was examined; participants were asked if they had ever smoked at least 100 cigarettes and, among those who had, how frequently on average they did or were doing so (i.e. daily, someday, or not at all). The individuals were classified as non-smokers, former smokers, occasional smokers, or daily smokers, according to the Centers for Disease Control and Prevention definitions^28^. All those identified as daily and occasional smokers (current smokers) were directed to complete the Fagerström Test for Nicotine Dependence (FTND). The fourth section (4) explored unhealthy alcohol consumption, using the Alcohol Use Disorders Identification Test (AUDIT) screening test. Lastly, in the fifth section (5) the item “How many days a week do you eat breakfast?” was used to assess breakfast skipping. The response options available ranged from 0 to 7 days a week, those who skipped breakfast for ≥ 3 days a week were classified as breakfast skippers.

#### Emotional intelligence

TMMS is a 30-item scale recognized to assess perceived EI that describes people's attitudes and beliefs about their own emotional experiences^29^. TMMS scores demonstrated high psychometric qualities and are robust to cross-cultural adaptations^30–34^ The original version of TMMS was previously back-translated to make sure that the Italian and English versions were highly accurate in terms of content and meaning by Giovannini et al*.*^35^. Moreover, it was validated in terms of internal consistency, factor structure, and concurrent validity^30^.

The scale assesses three domains of the emotional experience: attention to feelings, which is the tendency to notice emotional states; clarity of feelings, which refers to the capacity to identify and differentiate between different emotional states; and mood repair, which is the ability to regulate emotional states in order to better adapt to the situations. Items are assessed using a Likert-like scale of 5 points ranging from “Strongly disagree” (1) to “Strongly agree” (5). A score for each domain can be obtained; the total TMMS score provides a general, composite estimate of the examinee’s self-perceived EI.

#### Nicotine dependence

The Italian validated version of FTND^36^, a 6-item questionnaire, was applied to assess physical nicotine dependence among smokers. In scoring the FTND, 4 items are scored from 0 to 1 (yes/no items) and 2 items are scored from 0 to 3 (multiple-choice items). The four yes/no answers on the FTND are scored from 0 to 1, and the two multiple-choice items are scored from 0 to 3. A total score between 0 and 10 is generated after the items are added up. The higher the total FTND score, the more intense the individual's physical dependence on nicotine^37^.

#### Alcohol consumption

The AUDIT, developed by the WHO^38^, was used to explore alcohol consumption. This 10-item screening tool is considered a solid screening tool for hazardous alcohol use in university students^39–42^. The AUDIT has been translated to be used within the Italian population^43^. The first through eighth questions are scored on a five-point scale from 0 to 4, while questions nine and ten are scored on a three-point scale of 0, 2, and 4. Total score ranges from 0 to 40 points. A score of 8 or higher is considered a threshold indicative of problematic alcohol use, that increases the risk of harmful consequences for the user and is of public health significance.

### Ethical consideration

This study was approved by the Local Human Research Ethics Committee and was conducted in accordance with the Helsinki Declaration.

### Statistical analysis

All collected variables were summarized by means and standard deviations when normally distributed. Medians and IQR were used in cases of deviations from normality. The skewness of the variables was estimated by Shapiro–Wilk tests. Categorical variables were expressed as percentages. Univariate analyses were conducted to explore the relationship between EI domains and total score and nicotine dependence, alcohol consumption, and breakfast skipping. *T* tests were performed to determine if there was a significant difference in means if samples were normally distributed; the Wilcoxon-Mann–Whitney test was used if normality was violated. Furthermore, to provide a more accurate analysis, *p*-values have been adjusted using the Bonferroni correction to control for the number of comparisons. Cohen’s effect size was also measured. Multiple regression models were built to investigate the following outcomes of interest: being a current smoker (Model 1), medium–high nicotine dependence (Model 2), problematic alcohol consumption (Model 3), and breakfast skipping (Model 4). In all models, the variables age in years (continuous), gender (0 = male; 1 = female), majors attended (0 = medical or life science; 1 = social science or technology), and EI (TMMS total score, continuous) were included. Models 1, 2, and 3 included the variable alcohol consumption (no use/non-problematic drinking = 0; problematic drinking = 1). The variable breakfast skipping (no = 0; yes = 1) was included in Models 1, 2, and 4. The independent variable smoking status (non-smoker = 0; current smoker = 1) was included in Models 3 and 4. The statistical significance level was fixed at a *p*-value of < 0.05. Adjusted odds ratios (ORs) and 95% confidence intervals (CIs) were calculated. Statistical analysis was developed using Stata Statistical Software, Version 18^44^.

## Results

### General characteristics of participants

The socio-demographic characteristics of the study population and the prevalence of risky behaviors are detailed in Table 1. The study sample consisted of 537 participants, of whom 74.7% were women. The median age was 23 years (IQR: 20–25 years). More than half (59.8%) were enrolled in medical or life science courses.

**Table 1:** Socio-demographic characteristics and prevalence of risky behaviors among respondents.

Variables	Full sample (N = 537)	
	N	%
Gender		
Male	136	25.3
Female	401	74.7
Majors		
Medical or life science	321	59.8
Social science	148	27.6
Technology	68	12.6
Smoking status		
Undefined*	19	3.5
Never smokers	321	59.8
Former smokers	31	5.8
Current smokers (includes daily and occasional smokers)	166	30.9
Nicotine dependence^#^		
Low	134	80.7
Medium	29	17.5
High	3	1.8
Alcohol consumption		
No drinking	114	21.2
Non-problematic drinking	370	68.9
Problematic drinking^£^	53	9.9
Breakfast skipping		
≤ 2 days a week	413	76.9
≥ 3 days a week	124	23.1

*Those who responded “I don’t know” to question “Have you smoked 100 or more cigarettes in your life?”.

^#^Low: Fagerström score < 5; medium: Fagerström score from 5 to 7; high: Fagerström score > 7.

^£^AUDIT score ≥ 8.

### Tobacco use

Nearly a third of the sample were current smokers (23.8% were daily smokers; 7.1% were occasional smokers). According to the FTND, 19.3% of daily smokers were found to have moderate or high nicotine dependence. No significant differences were found between the EI levels of current smokers and non-smokers. Emotional clarity and total EI scores resulted significantly lower in current smokers with moderate or high nicotine dependence than those with low nicotine dependence, with medium effect size (d = 0.55 and d = 0.59, respectively) (Table 2). Results of the multiple logistic regression analysis indicated that risky alcohol consumption (OR: 3.36; 95% CI 1.78–6.38) and breakfast skipping (OR: 2.90; 95% CI 1.85–4.57) were significant predictors of being a current smoker (Model 1 in Table 3). Moreover, the odds of having high nicotine dependence were significantly lower among those with better EI levels (OR: 0.95; 95% CI 0.92–0.98), among younger (OR: 1.26; 95% CI 1.08–1.46) and among students attending to medical or life science majors compared with those attending to social science or technology majors (OR: 2.51; 95% CI 1.02–6.16) (Model 2 in Table 3).

**Table 2:** Mean differences in EI domains and total score according to nicotine dependence, alcohol consumption and breakfast skipping.

	Low dependence nicotine n = 134	Medium–high dependence n = 32	*p*-value unadjusted	Bonferroni adjustment*	Cohen’s *d*
EA	49.8** ± **6.9	47.2** ± **7.3	0.065	0.065	0.36
EC	36.8** ± **7.4	32.6** ± **8.5	**0.005**	**0.005**	0.55
ER	19.7** ± **5.3	17.6** ± **4.8	**0.042**	0.042	0.40
Total	106.3 ± 14.8	97.4 ± 15.7	**0.003**	**0.003**	0.59

	Non problematic drinking n = 484	Problematic drinking n = 53			
EA	49.1 ± 7.2	47.7** ± **7.3	0.082	0.169	0.20
EC	37.2 ± 8.0	33.7** ± **8.1	**0.003**	**0.003**	0.43
ER	19.6 ± 4.8	18.0** ± **5.9	**0.045**	0.027	0.32
Total	105.8 ± 14.4	99.3 ± 15.5	**0.004**	**0.002**	0.45

	Breakfast eaters n = 413	Breakfast skippers n = 124			
EA	49.5** ± **6.8	47.2** ± **8.0	**0.002**	**0.002**	0.33
EC	37.2** ± **7.6	35.5** ± **9.3	**0.003**	0.030	0.22
ER	19.7** ± **4.7	18.5** ± **5.5	0.054	0.022	0.23
Total	106.4 ± 13.6	101.2 ± 17	**0.002**	** < 0.001**	0.36

*EA* emotional attention, *EC* emotional clarity, *ER* emotional repair.

*Bonferroni correction, p < 0.012.

Significant values are in bold.

**Table 3:** Results of the regression models for potential predictors of the outcomes of interest.

Model 1 Outcome: being current smoker Log likelihood = − 289.89338; Prob > chi2 < 0.001; Obs = 487^§^			
Variables	OR	95% CI	*p*-value
Alcohol consumption			
No use/non problematic drinking*	1.00		
Problematic drinking	3.36	1.78–6.38	< 0.001
Breakfast skipping			
No*	1.00		
Yes	2.90	1.85–4.57	< 0.001
Gender			
Male*	1.00		
Female	0.80	0.51–1.25	0.330
Emotional intelligence			
TMMS total score, continuous	1.00	0.99–1.02	0.413
Majors attended			
Medical or life science*	1.00		
Social science or technology	1.04	0.69–1.56	0.855
Age in years, continous	1.00	0.93–1.06	0.909

Model 2 Outcome: medium–high nicotine dependence Log likelihood = − 67.460028; Prob > chi2 = < 0.001; Obs = 166^#^			
Variables	OR	95% CI	*p*-value
Emotional intelligence			
TMMS total score, continuous	0.95	0.92–0.98	0.001
Age in years, continous	1.26	1.08–1.46	0.003
Majors attended			
Medical or life science*	1.00		
Social science or technology	2.51	1.02–6.16	0.045
Gender			
Male*	1.00		
Female	2.54	0.87–7.41	0.088
Breakfast skipping			
No*	1.00		
Yes	2.07	0.85–5.02	0.158
Alcohol consumption			
No use/non problematic drinking*	1.00		
Problematic drinking	0.58	0.17–1.94	0.377

Model 3 Outcome: problematic alcohol consumption Log likelihood = − 142.77706; Prob > chi2 < 0.001; Obs = 487£			
Variables	OR	95% CI	*p*-value
Smoking status			
Non-smoker*	1.00		
Current smoker	3.42	1.81–6.46	< 0.001
Breakfast skipping			
No*	1.00		
Yes	2.29	1.20–4.35	0.012
Gender			
Male*	1.00		
Female	0.49	0.26–0.93	0.030
Emotional intelligence			
TMMS total score, continuous	0.98	0.96–1.00	0.050
Majors attended			
Medical or life science*	1.00		
Social science or technology	1.56	0.83–2.92	0.164
Age in years, continous	0.98	0.89–1.09	0.752

Model 4 Outcome: breakfast skipping Log likelihood = − 240.83305 ; Prob > chi2 < 0.001; Obs = 487£			
Variables	OR	95% CI	*p*
Smoking status			
Non-smoker*	1.00		
Current smoker	2.89	1.83–4.54	< 0.001
Alcohol consumption			
No use/non problematic drinking*	1.00		
Problematic drinking	2.27	1.19–4.35	0.013
Emotional intelligence			
TMMS total score, continuous	0.98	0.97–0.99	0.013
Age in years, continous	0.93	0.86–1.00	0.064
Majors attended			
Medical or life science*	1.00		
Social science or technology	0.76	0.48–1.20	0.237
Gender			
Male*	1.00		
Female	0.89	0.54–1.47	0.652

*Reference category.

^§^Former smokers were excluded.

^#^Observation included only current smokers.

### Alcohol consumption

About eight out of ten (78.8%) consumed alcohol at least once in their lifetime. The mean AUDIT score was 3.07 (SD ± 3.34) and problematic drinking (AUDIT score ≥ 8) was shown in 9.9% of the sample. Individuals with problematic alcohol consumption showed significantly lower emotional clarity and total EI scores than those with AUDIT score < 8 and the effect size resulted medium (d = 0.43 and 0.45, respectively) (Table 2). Furthermore, the results of Model 3 highlighted that the strongest predictor of problematic alcohol consumption was being a current smoker (OR: 3.42; 95% CI 1.81–6.46). Other predictors were breakfast skipping (OR: 2.29; 95% CI 1.20–4.35) and being male (OR:0.49; 95% CI 0.26–0.93) (Table 3).

### Breakfast skipping

Almost one-fourth (23.1%) reported breakfast skipping ≥ 3 days a week. Breakfast skippers showed significantly lower emotional attention and global EI scores than breakfast eaters, with medium effect size (d = 0.33 and d = 0.36, respectively) (Table 2). Results of the multiple logistic regression analysis showed that being a current smoker (OR: 2.89; 95% CI 1.83–4.54) and making problematic alcohol use (OR: 2.27; 95% CI 1.19–4.35) were significant predictors of skipping breakfast (Model 4, Table 3). Moreover, each one-point increase in total EI score resulted in a 2% reduction in the odds of breakfast skipping (OR: 0.98; 95% CI 0.97–0.99) (Model 4 in Table 3).

## Discussion

To the best of our knowledge, this study represents one of the first attempts to explore the relationship between EI and unhealthy lifestyles, namely tobacco use, nicotine dependence, alcohol consumption, and breakfast skipping among university students. According to the majority of theoretical models of decision-making, emotions play a significant role in the mechanism that directs individual behavior in risky situations and forms his/her lifestyle^45–47^. Lifestyle has a significant influence on the physical and mental health of human beings. Millions of people today adopt unhealthy lifestyles, which can lead to illness, disability, and even death^48^.

A major result of this survey is the lack of significant EI differences between smokers (current smokers) and non-smokers, as shown in a previous study^49^. However, in contrast to published evidence^50,51^, EI levels appear to be related to the degree of nicotine dependence measured by FTND. Indeed, higher levels of EI seem to protect smokers from moderate/high nicotine dependence. This figure is in line with neurobiological studies regarding nicotine dependence and stress reactivity. Grabowska et al. have highlighted how dependence, once established, could become a source of stress and how chronic stress can feed addiction^52^. Low EI skills could make it even more difficult to break this vicious circle. This is a cause for concern since nicotine addiction is a complex disorder that is challenging to treat and continues to have a major impact on health all over the world^53^. According to the latest Italian National Institute of Health report the prevalence of smokers in the Italian population in 2022 was 24.2%^5^. Young adults should be a primary target for smoking prevention and cessation programs^54^, and EI should have as much of a focus as cognitive intelligence. Emotional learning programs among adolescents (e.g. in schools) could be a worthwhile investment to protect individual health and promote better public health outcomes later in life^55^. Also being enrolled in a medical or life science course seems to play a protective role from developing a moderate/high nicotine dependence. It could be argued that the exposure to health-related topics may lead to a better understanding of risks and help those students to establish more awareness of smoking consequences than other students^56^ and to make healthier life choices.

Lower EI levels are also linked to risky alcohol consumption, in line with a previous study^57^. Although the prevalence of risky alcohol consumption seems contained in the sample (9.9%), attention needs to be kept high since drinking is responsible for 14% of young deaths^58^. It is known that a significant percentage of high-risk drinkers consume alcoholic beverages to manage negative emotions^59^, and the neural pathway of such behavior was characterized^60^. As clarified by Barbey et al., damage to the neural EI network can affect the ability to manage emotions, and the evidence from the present study suggests an opportunity to intervene on the issue^61^. It could be argued that enhancing EI could improve the management of emotions which might help reduce the likelihood of alcohol consumption^62^, as well as unhealthy lifestyles. Individuals exhibit unique emotional control mechanisms throughout their lives. Consistent patterns of affective responding, social relationships, and even overall life satisfaction have been linked to these distinctive patterns of habitual emotion regulation usage^63^. According to recent research, individual differences in the way people regulate their emotions may have neural correlates that can be seen in the way they unconsciously process emotional stimuli^63^. The process of such changes within the brain is referred to as ‘neuroplasticity’. Thanks to neuroplasticity, the brain has the ability to adapt and reorganize itself based on experiences and behaviors. EI is important to this process to effectively regulate emotions and respond to emotional stimuli in a balanced manner^64^.

Since unhealthy eating behavior was linked to considerable deaths worldwide, we decided to investigate breakfast skipping, as a proxy measure of undesirable diet. We hypothesized that individuals who skip breakfast may be more likely to eat excessively or consume unhealthy foods throughout the day, leading to an increased risk of obesity and chronic illnesses, such as diabetes and cardiovascular diseases. Indeed, a recent meta-analysis indicated an 11% increased RR for overweight/obesity when breakfast was skipped on ≥ 3 days per week compared to ≤ 2 days per week^65^. The survey findings pointed out a high prevalence (23.1%) of this practice, and a novel association with EI, suggesting that breakfast skipping could have broader implications for overall well-being. Given that people with low EI have worse emotion regulation than people with high EI, and that poor emotion regulation can result in problematic eating behaviors linked to weight gain^66^, the latter study finding represents an important step towards deepening the understanding of mechanisms underlying unhealthy dietary behaviors. Additionally, the study revealed that individuals who skipped breakfast were more likely to engage in unhealthy behaviors such as smoking and alcohol abuse, further emphasizing the need to address this issue as part of public health interventions. Further research is needed to understand the underlying mechanisms and develop tailored interventions to promote healthy breakfast habits and improve both physical and emotional well-being.

Last but not least, the risky behavior combination occurs more frequently than would be estimated if they were independent. Indeed, both drinking alcohol and skipping breakfast predicted current smoking, just as being a current smoker predicted problematic drinking and breakfast skipping. Once again, the results demonstrated how unhealthy behaviors are interconnected and how one behavior may influence or predict another^67^. These data reinforce that the co-occurrence of multiple unhealthy factors is a widespread problem^68,69^, and the complex interplay between different behaviors when developing public health strategies and policies must be acknowledged, as well as that interventions simultaneously targeting multiple risk behaviors may be more effective in promoting overall health and well-being. In the meta-analysis conducted by Meader et al. to explore the effectiveness of multiple risky behavior interventions, the authors concluded that non-pharmacologic interventions, i.e., education and skills training, resulted in small reductions in unhealthy behaviors and meaningless reduction in risk of overall mortality^70^. In this context, the need arises to develop innovative integrated approaches impacting lifestyle choices. It may be essential for achieving this goal that these approaches take into account the unconscious mechanisms underlying the discrepancy between knowledge and practice. Since knowledge is not sufficient to make healthy choices^71,72^, privileging the role of information as the most important driver of behaviors could represent a missed opportunity for public health. Although EI training has primarily been used in leadership programs, good results have been achieved implementing EI training interventions aimed at increasing well-being. In particular, the Italian National Institute of Health has developed a handbook to improve EI among high school students^73^ and its implementation has proven effective in promoting health^74^. Persich et al. developed an empirically based, online training program to enhance EI, that appeared to be effective at sustaining critical aspects of mental health during the COVID-19 pandemic^75^.

To appreciate the findings of this study, some limitations must be acknowledged. First, the cross-sectional design of the study does not allow to conclude causality about the observed associations. However, the present research allows us to assess unhealthy lifestyles and EI among university students, which will be useful for public health interventions and help the scientific community develop hypotheses regarding the influence of EI on the tendency to engage in risky behaviors or to promote healthy choices. Second, the data were self-reported and collected through an electronic self-administered questionnaire and thus potentially subjected to bias such as recall and social desirability bias. Nonetheless, these biases were mitigated as the methodology of collection assured the anonymity of the participants and avoided the possible influence of the presence of an interviewer on the respondent's answers. Additionally, a self-administered questionnaire, in which the respondent has the majority of control and may read the questions and answer at their own pace, may yield more trustworthy and consistent results. Third, the choice of the most appropriate measure of EI for a specific purpose is still a topic of debate in the scientific community^30,76^. The TMMS used in the present study to assess EI was a self-report instrument rather than a performance-based tool. Although performance-based EI measures are often considered preferable to self-report measures, the TMMS represents an easier instrument to collect data with, does not require any training to be used, and produces a reliable assessment of EI^30^. Indeed, the TMMS scores have shown good psychometric properties and have proven to be valid measures of EI in several studies^34,77,78^. Finally, since the study sample was restricted to students at a single university in Southern Italy, it is important to use caution when generalizing the results to the entire community of Italian university students.

## Conclusions

The study findings provide useful insights to develop evidence-based integrated strategies to empower the young adults to choose a health-promoting lifestyle. The figures suggest that emotional learning interventions could support this goal. Future research should focus on evaluating the effectiveness of different emotional learning interventions in diverse populations and settings, as well as on exploring the long-term impact of these interventions on overall well-being. If prospective studies would confirm the hypothesis that increasing EI may reduce unhealthy lifestyles, it will have significant implications for health promotion programs.

## Data Availability

The dataset generated by the survey research and analyzed during the current study is available in the Mendeley Data repository, 10.17632/xwjn9t2grz.1.
